# Seroprevalence of Infections with TORCH Agents in Romania: A Systematic Review

**DOI:** 10.3390/microorganisms11082120

**Published:** 2023-08-20

**Authors:** Cristiana Luiza Radoi, Ovidiu Zlatian, Maria Balasoiu, Lucian Giubelan, Andreea Cristina Stoian, Livia Dragonu, Alexandru Neacsu, Dominic Gabriel Iliescu

**Affiliations:** 1 Doctoral School, University of Medicine and Pharmacy of Craiova, 200349 Craiova, Romania; luizacristianaradoi@gmail.com (C.L.R.); alex.neacsu1210@gmail.com (A.N.); 2 Medical Laboratory, County Clinical Emergency Hospital of Craiova, 200349 Craiova, Romania; balasoiu.maria@yahoo.com; 3 Microbiology Department, University of Medicine and Pharmacy of Craiova, 200349 Craiova, Romania; 4 Infectious Diseases Department, University of Medicine and Pharmacy of Craiova, 200349 Craiova, Romania; ligiubelan@yahoo.com (L.G.); andreea_plr@yahoo.com (A.C.S.); livia_dragonu@yahoo.com (L.D.); 5 “Victor Babes” Infectious Diseases and Pneumophtisiology Clinical Hospital, 200515 Craiova, Romania; 6 Obstetrics and Gynecology Department, County Clinical Emergency Hospital of Craiova, 200349 Craiova, Romania; dominic.iliescu@yahoo.com; 7 Obstetrics and Gynecology Department, University of Medicine and Pharmacy of Craiova, 200349 Craiova, Romania

**Keywords:** TORCH agents, antibodies, seroprevalence, childbearing age women, Romania

## Abstract

Maternal–fetal infectious pathology—notably the TORCH panel (*Toxoplasma gondii*, rubella, Cytomegalovirus, and herpes simplex viruses)—critically impacts maternal and neonatal health. This review collates data on the seroprevalence of IgG and IgM antibodies against TORCH agents in Romanian women, aiming to discern regional and population differences and identify risk factors. Twenty studies were included in the review, revealing variable seroprevalence rates across the country. Regions such as Moldavia and Banat showed higher anti-*T. gondii* IgG seroprevalence rates than Bihor, with notable declines in Banat. Rural, older, and multiparous women showed elevated *T. gondii* IgG rates. Anti-rubella vaccine introduction significantly reduced the prevalence of anti-rubella IgG antibodies, but recent vaccination coverage decreases raise concerns. CMV and HSV seroprevalence varied geographically, with rural areas generally showing higher CMV rates and HSV influenced by factors like education level and number of sexual partners. Concurrent seroprevalence of multiple TORCH components in some cases underscores potential common risk factors. This study highlights the importance of continuous monitoring and preventive measures such as vaccinations and awareness campaigns to mitigate the health impact on the pregnant population.

## 1. Introduction

Maternal–fetal infectious pathology is a crucial subject in the fields of obstetrics–gynecology and neonatology, as it directly impacts the health and wellbeing of both mother and child [1]. These infections are more frequent in immunodeficient patient groups, such as those with organ transplants, HIV infection, and other immunocompromising diseases [2,3]. This research emphasizes the significance of serological testing for infectious agents encompassed in the TORCH panel, which includes *Toxoplasma gondii* (*T. gondii*), rubella virus, Cytomegalovirus (CMV), and herpes simplex viruses (HSVs) type 1 and 2. These tests are particularly relevant for women who are attempting to conceive, those who are already pregnant and, in certain situations, their newborns [4].

Serological analyses for these infectious agents can identify infections with microorganisms known to negatively affect the course of a pregnancy, the health of the developing fetus, and the subsequent wellbeing of the newborn. Early detection of these infections is vital, as they have been associated with a range of adverse pregnancy outcomes, including miscarriages, preterm births, and fetal or congenital malformations in the newborn [4,5].

By identifying and addressing these infections early in the pregnancy or preconception period, healthcare providers can implement appropriate interventions to minimize risks and promote healthier outcomes for both mother and child [1,4]. Furthermore, understanding the prevalence and distribution of these infectious agents within specific populations can help guide public health initiatives aimed at reducing the overall burden of maternal–fetal infectious diseases, can emphasize the need for funding for public health interventions, and can aid in healthcare planning for reducing the burden of TORCH infections [6].

### 1.1. Toxoplasma gondii

*Toxoplasma gondii* is a globally distributed protozoan parasite responsible for causing toxoplasmosis in humans and warm-blooded animals. Its worldwide prevalence varies significantly between countries and regions, with an estimated one-third of the global human population carrying a *T. gondii* infection [7].

The global epidemiology of *T. gondii* infection is influenced by a multitude of factors, such as environmental conditions, food habits, and socioeconomic factors [7]. Some of the highest prevalence rates are found in Latin America (50–80%) [8,9], while the Middle East (30–40%) [10], Southeast Asia (20–30%) [11], and Africa (40–70%) show moderate-to-high prevalence rates [12]. The lowest prevalence rate (13.3%) was found in the USA in large studies [13]. Factors contributing to this variation include differences in climate, population density, cat ownership, dietary habits, and hygiene practices [9,14].

In some Eastern European countries like Croatia [15] and Bosnia–Herzegovina [16], the seroprevalence rates in blood donors tend to be higher (52.51% and 30.6%), possibly due to more frequent consumption of raw or undercooked meat and contact with infected soil [17]. In Serbia, another country in Eastern Europe, the seroprevalence of anti-*T. gondii* IgG antibodies in blood donors was reported to be 20.5% [17].

### 1.2. Rubella Virus

Globally, the incidence of rubella has decreased significantly over the past few decades, mainly due to the widespread use of the measles, mumps, and rubella (MMR) vaccine [18]. According to the World Health Organization (WHO), in 2000, there were an estimated 670,000 cases of rubella, while in 2019, the number of reported cases was only around 12,000 [19]. In 2012, the WHO set a goal to eliminate rubella and CRS (congenital rubella syndrome) from at least five of the six regions (Africa, Americas, Eastern Mediterranean, Europe, Southeast Asia, and Western Pacific) by 2020 [20].

In Europe, the epidemiology of rubella has improved substantially due to the widespread implementation of vaccination programs. A study performed by the WHO showed that between 2005 and 2009 the number of rubella cases in the European region decreased by 94% (if in 2005 there were 206,359 cases, in 2009 the number had decreased to 11,623). A high incidence was recorded in Italy, San Marino, Poland, Bosnia–Herzegovina, and Austria. In the same period, only 68 CRS cases were reported [21]. Another WHO report mentions a decline in median incidence from 7.2 in 2000 to 0.3 in 2008. Between 2000 and 2008, 21,475 rubella cases were reported to the WHO, mostly from Italy, Poland, and Romania [22]. In 2012, in the European region, 29,601 rubella cases were reported by the WHO, while in 2013 39,367 cases were reported [23]. In 2018, the European Regional Verification Commission for Measles and Rubella Elimination (RVC) verified that 35 of the 53 countries in the WHO’s European region had eliminated rubella. However, sporadic outbreaks continue to occur in some countries where vaccination coverage is insufficient, particularly in Eastern Europe [24].

In the epidemic outbreak in Europe from 2011 to 2012, the age group most affected was 15 to 19 years—unvaccinated teenagers [25]. The prevalence of acute rubella was shown to be higher in urban areas [26]. In 2020, the WHO reported an incidence rate of acute rubella infection in Europe of 0.3/1.000.000 people [18].

### 1.3. Cytomegalovirus

Among women of reproductive age in Japan, the seroprevalence of anti-CMV IgG antibodies was estimated as 60.2%, as opposed to 58.3–94.5% in Latin America, 24.6–81.0% in North America, and 45.6–95.7% in Europe [27]. The CMV IgG seroprevalence across Europe ranged from 57.3% in Poland [28] to 95.7% in Romania [29,30]. In Northern and Western Europe, CMV IgG seroprevalence varied between 45.6 and 65.9% [31,32,33].

### 1.4. Herpes Simplex Viruses

According to a meta-analysis by James et al., 1816.5 million women (66.1%) were infected with oral HSV-1 and 92.5 million women (5.1%) with genital HSV-1, while 313.5 million (17.1%) women aged between 15 and 49 years were infected with HSV-2 [34].

The seroprevalence of anti-HSV IgG antibodies in the United States between 1999 and 2010 was 53.0% for HSV-1 and 15.7% for HSV-2 [35]. Later, between 2015 and 2016, another study from the United States reported a seroprevalence of HSV-1 of 48% among 14–49-year-olds, while HSV-2 seroprevalence was 12% [36].

In Asia, the seroprevalence of HSV-1 has been reported to be between 70% and 90% in various countries [37,38]. HSV-2’s seroprevalence in Asia varies widely, ranging from 5% to 30% [38]. The seroprevalence of HSV-1 in Africa is high, with most studies reporting rates above 80% [39]. HSV-2’s seroprevalence is also high in Africa, with estimates ranging from 30% to 80% in various populations [39,40]. In Latin America, the seroprevalence of HSV-1 ranges from 60% to 90%, while HSV-2’s seroprevalence varies from 10% to 40% across different countries [41].

The seroprevalence of HSV-1 in Europe varies, ranging from 52% in the United Kingdom to 84% in Bulgaria [42]. A meta-analysis published in 2020 reported a mean seroprevalence of anti-HSV-1 IgG of 69.5% among women, and by region 78.7% in Eastern Europe, 77.2% in Southern Europe, 61.6% in Western Europe, and 57.7% in Northern Europe [43].

HSV-2’s seroprevalence in Europe is generally lower, with estimates ranging from 4% to 24% [42,44,45,46]. Another study using mathematical modeling indicated that in Europe, in 2016, 10.7% of females and 5.3% of males aged 15–49 years were infected with HSV-2 [47].

A meta-analysis study performed using European studies reported a mean seroprevalence of anti-HSV-2 IgG of 14.0% in women, and by region the seroprevalence was 9.6% in Eastern Europe, 12.2% in Southern Europe, 13.2% in Western Europe, and 13.5% in Northern Europe [47].

## 2. Aims and Scope of this Review

To date, for the majority of EU countries, there is sparse population-based prevalence data of TORCH infections in women. Moreover, as far as we are aware, these prevalence rates have rarely been summarized at the global, regional, or national levels. We could not find any systematic review or meta-analysis of the prevalence rates of these infections in Romania. However, there is a growing interest in the benefits of TORCH panel antibody screening in pregnant women, given the low number of acute infections detected. Consequently, our objective was to review and synthesize the known data about the seroprevalence of IgG and IgM antibodies against agents of the TORCH complex in women from Romania, and to highlight the differences in different population groups within the country. Also, this review aimed to analyze the impact of various risk factors on the seroprevalence of these antibodies, which could improve existing prevention strategies. Additionally, we evaluated the geographical distribution and population-specific prevalence rates of TORCH agents in Romania, identifying significant trends or patterns in the data.

The current review can provide a comprehensive overview of the seroprevalence of TORCH agents in Romania, which can assist in future research, public health initiatives, and clinical practice.

## 3. Methods

The systematic review methodology was in accordance with the Preferred Reporting Items for Systematic reviews and Meta-Analyses (PRISMA) guidelines.

The inclusion criteria for studies used in this review were as follows:

- Studies that report the seroprevalence of at least one TORCH agent (*T. gondii*, Rubella virus, CMV, and HSV-1/2) in the general population of Romania or a specific population group within Romania, particularly women.
- Studies published in the English or Romanian language.
- Studies with available data on sample size, age range, and seroprevalence rates for each TORCH agent.
- Studies published up to February 2023.

The exclusion criteria used were as follows:

- Studies published in languages other than English or Romanian.
- Articles that were duplicates, editorials, or opinion pieces without original data.
- Studies without human subjects.

**Figure 1:**
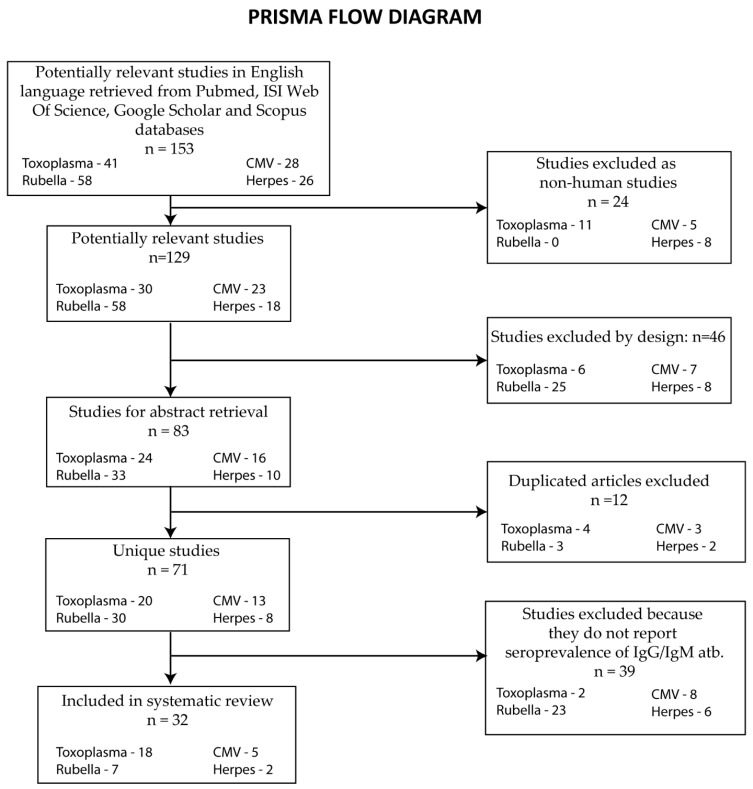
PRISMA flow diagram of the study selection process.

To investigate the seroprevalence of infections with TORCH agents in Romania, we conducted a systematic review of relevant studies using the following search strategy: We searched the following electronic databases: PubMed, Google Scholar, Scopus, and ISI Web of Science. The search phrases used were in the form “Agent” AND “seroprevalence” AND “women” AND “Romania”, where “Agent” was replaced with “Toxoplasma”/“Rubella”/“CMV”/“HSV”. Another strategy was to search for the diseases produced by these agents, e.g., “toxoplasmosis”. The search was limited to studies published in the English or Romanian language up to February 2023. The search yielded a total of 153 titles (41 *T. gondii* + 58 rubella + 28 CMV + 26 herpes simplex). After excluding the non-human studies and articles by design, 83 studies remained. After excluding 12 duplicates, we screened the abstracts of the remaining 71 studies. After reading the abstracts and full texts, we excluded 39 additional studies that did not report the prevalence. Finally, we included 32 articles in our review, and we extracted relevant data from each study, including sample size and seroprevalence rates for IgG and IgM antibodies against agents of the TORCH complex. This selection process is presented in the PRISMA flow diagram (Figure 1).

### Quality Assessment

This review was founded upon observational studies, inherently carrying a risk of bias. Factors that may compromise the comparability and transferability of the study outcomes include discrepancies in the study populations, variations in sample size, divergent observation periods, distinct demographic regions, and disparate research methodologies.

Furthermore, our search for eligible studies was restricted to the PubMed, Google Scholar, ISI Web of Science, and Scopus databases, potentially neglecting relevant investigations available in alternative databases. Due to these considerations, this systematic review is not exempt from bias, rendering the interpretation of its findings constrained.

## 4. Results

In Romania, the majority of studies about the seroprevalence of antibodies against TORCH agents were performed after 1989 [48,49], immediately after the reagents needed to detect the antibodies became available after the fall of communism in Romania [50,51,52].

### 4.1. Toxoplasma gondii

#### 4.1.1. Anti-*Toxoplasma gondii* IgG

The seroprevalence in the Romanian general population varied from region to region. A large study performed in 2009 on healthy people from 11 counties from northwestern and central Romania [53] reported that the seropositivity rate was 59.48% in the general population, while in western Romania a seroprevalence rate of 64.8% was reported in 2015 [54]. In healthy blood donors—a group that can estimate the true population prevalence of anti-*T. gondii* IgG antibodies—Lupu et al. [55] reported a seroprevalence of anti-*T. gondii* IgG of 45.9% in western Romania in 2022.

In pregnant women, we found one study performed in 1998 reporting a seropositivity for *T. gondii* IgG of 43.9% in northeast Romania [50], while in central Romania in 2008 the seroprevalence was 39.0% in a study performed in 510 pregnant women [56] and 38.24% [57] in 2018 in another study from the Muntenia region. In western Romania, the seroprevalence was 55.8% [58]. A study by Motoi et al. showed a descending trend of seroprevalence from 43.79% in 2008–2010 to 38.81% in 2015–2018 [59]. In the Bihor area of western Romania, a study by Csep et al. reported a lower seroprevalence of 25.4% in 2022 [60].

In women of reproductive age, a study performed in 2021 in northeast Romania (Moldavia region) found a seroprevalence of anti-*T. gondii* IgG of 33.84% [61]. In southeast Romania (Dobrogea region), the seroprevalence was 33.16% [14]. In a sample of 105 women aged between 16 and 35 years from northwest and central Romania, the seroprevalence was determined to be 51.43% [53]. In western Romania, the seroprevalence rate was reported at 57.6% in 2008 [62], 55.55% in 2015 [54], and 41.16% in 2020 [63]. In the same region, a descending trend of seroprevalence was reported from 43.7% in 2008–2010 to 38.8% in 2015–2018 [30]. Future studies should confirm this descending trend. In reproductive-age female blood donors from western Romania in 2022, a seroprevalence rate of 43.2% was reported [55]. In the Bihor region, a lower seroprevalence of 36.48% was reported in 2022 [64].

A study performed in northwest Romania in children with mental retardation and visual pathology showed a seroprevalence of IgG antibodies of 66.4% and 37.4%, respectively, compared with the seroprevalence rate of only 9.3% in healthy children [50]. In a sample of 15 children from southeast Romania, the seroprevalence was 0% for IgM antibodies and 33.33% for IgG [14]. Another study from western Romania reported a seroprevalence rate of 16.66% in children with pathologies unrelated to *Toxoplasma* infection [65]. These seroprevalence figures in children suggest *Toxoplasma* exposure since childhood, since the screening should not be limited to pregnant women but extended to the child population.

However, these data must be interpreted with caution, as the study groups used were from different institutions: one from a public hospital and one from a private laboratory. People addressing these two types of institutions may have different population structure in terms of age and education level, for example, and previously a higher seroprevalence of anti-*Toxoplasma* IgG antibodies was reported in women of reproductive age with lower educational status [66].

#### 4.1.2. Risk Factors

Regarding the sex prevalence, there were no significant differences between males (60.4%) and females (68.5%) [54]. The seroprevalence of IgG antibodies generally increased with age, as showed by Olariu et al. [62], higher in women aged over 40 years (71.4%) compared with young women under 20 years of age (33.3%). This difference was confirmed by studies performed 7 years later [54] and 12 years later in the Banat region by Motoi et al. and Mihu et al., respectively [59,63,64]. The estimated annual infection risk associated with age showed high susceptibility from childhood to adolescence (3–4.5% annual infection risk), a stable level for adults (1–2%), and then an increase up to 5.5% for individuals over 65 years old [53]. A recent study performed on healthy blood donors [55] showed that seroprevalence also increased with age, from 32.6% in the 18–25 years age group to 67.6% in the 56–63 years age group. The seroprevalence increased as the education level decreased. The multivariate analysis detected significant associations between seroprevalence and owning pets, age, and education level [55].

There are more possible explanations for the observed decrease in the seroprevalence of *T. gondii* infection. Cat owners, especially in urban areas, feed them with canned cat food, which is sterilized. Fewer cats are allowed to hunt rodents, which are the source of infection with *Toxoplasma* [67]. The raw meat that is commercially available is generally *Toxoplasma*-free, as the meat is deep-frozen during transportation and the cold inactivates *Toxoplasma* [55,68]. A study from Romania performed on pregnant women found a significant association between toxoplasmosis and the consumption of undercooked meat (*p* < 0.05) [57].

Vegetables are generally washed, which may decrease contamination with oocysts. Lastly, drinking water quality has improved, and more people consume bottled drinking water, which is free of oocysts [68]. Rugina et al. (2015) performed a multivariate analysis in a study on women from the Dobrogea region that showed risk ratios of 3.27 for consumption of raw/uncooked meat, 2.47 for unpasteurized milk, and 2.23 for tasting meat during cooking [14].

**Figure 2:**
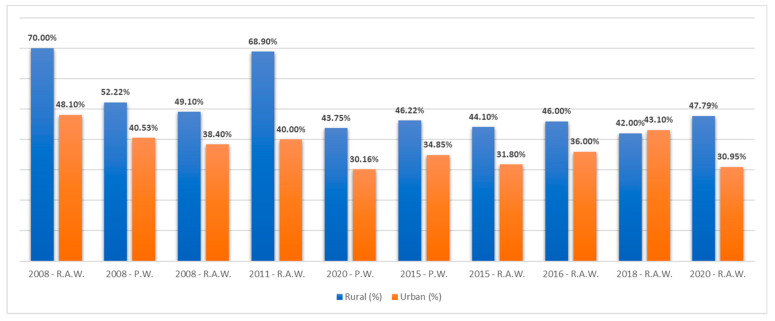
Differences in prevalence of anti-*T. gondii* IgG antibodies between rural and urban areas. P.W.: pregnant women; R.A.W.: reproductive-age women [30,54,55,58,59,62,63].

Furthermore, variations in prevalence were noted in relation to the residential area. The prevalence in urban areas was lower (55.3%) compared with rural areas (76.9%) [54]–a difference also noted by Mihu et al. (36% vs. 46%) [63,64] (Figure 2).

The seroprevalence of *T. gondii* was observed to be higher among pet owners (specifically, cats and/or dogs) compared to individuals without pets (*p* = 0.032) [58]. Also, females with a history of four or more live births exhibited a higher frequency of *T. gondii* seropositivity than those who had no previous births (*p* < 0.002) [58].

#### 4.1.3. Anti-*Toxoplasma gondii* IgM

In the Banat region (Table 1), a prevalence of anti-*T. gondii* IgM antibodies of 0.821% was reported between 2008 and 2010, which increased in 2015–2018 to 1.054% [30]. In a study from the Bihor area that included pregnant women addressed to the infectious disease clinics [60], there was an extremely high prevalence of 14.6% of anti-*T. gondii* IgM, but there was a strong selection bias, as these pregnant women were referred because they presented clinical signs of toxoplasmosis. Recently, Mihu et al. [66] reported a prevalence of IgM antibodies of 8.90% in females of reproductive age. The prevalence of anti-*T. gondii* IgM antibodies could not be related to age group (*p* = 0.0997), place of residence (*p* = 0.3105), or year of study (*p* = 0.4410) [60].

In the Moldavia region, a study reported a seroprevalence of 7.28% of anti-*T. gondii* IgM [61]. Of the 15 IgM-positive women, 10 were in the 25–34 years age group, 4 in the 35–44 years age group, and 1 in the 15–19 years age group [61].

In the Dobrogea region, a seroprevalence of 2.46% of IgM antibodies was reported in women [14]. A study from the Transilvania region [56] reported a seroprevalence of IgM antibodies of 1.76% in pregnant women between 2005 and 2007. The method used to detect IgM antibodies was manual ELISA. Acute toxoplasmosis infection was predominantly observed during the first trimester of pregnancy, accounting for 66.66% of cases and representing the primary cause of abortion. The annual infection risk for women aged 20–33 years in this geographic region was determined to be 0.67%. Around 4% of cases displayed IgM persistence for over a year, while an additional 7% exhibited both positive IgM and positive IgG, suggesting a potential persistence in 11% of cases [56].

As for the Muntenia region, a study performed on women in Bucharest found a seroprevalence of 17.65% for anti-*T. gondii* IgM And 38.24% for anti-*T. gondii* IgG [57]. Out of the 30 women who were positive for both IgM and IgG, only 13.3% presented all indications (positive specific IgM and IgA antibodies, low IgG avidity, and positive PCR) for maternally acquired *T. gondii* infection during pregnancy, posing a risk of congenital transmission.

In interpreting the results of the studies regarding IgM antibodies, we must consider that acute toxoplasmosis can result in the persistence of anti-*T. gondii* IgM antibodies for several years, so the IgM-positive patients cannot be regarded as acute toxoplasmosis cases.

### 4.2. Rubella Virus

#### 4.2.1. Anti-Rubella IgG

The prevalence of anti-rubella IgG antibodies was heavily influenced by the introduction of anti-rubella vaccines. The bivalent measles–rubella vaccine was first administered in Romania in 1998 to girls aged 15–18 years (born between 1980 and 1983); the introduction of this vaccine occurred following a measles outbreak. In April 2003, rubella vaccination was introduced for 13–14-year-old girls (eighth-grade students) during school-based campaigns. In May 2004, the monovalent measles vaccine was replaced in the national vaccination program with the trivalent measles–mumps–rubella (MMR) vaccine for children aged 12–15 months, with plans to introduce it to first-grade children (7 years old) in 2005. During this period, rubella vaccination continued for eighth-grade girls (13–14 years old) until 2008 [70,71].

The first study that we found reporting anti-rubella IgG prevalence in Romania was from 1989 (Table 2), performed on 5030 women aged 15–40 years, tested by hemagglutination inhibition reaction. In this study, 76.7% of sera were reactive for IgG antibodies [49]. Between 2002 and 2003, a large rubella outbreak of 115,000 cases was reported in Romania, with an incidence rate of 531/100,000 inhabitants and 150 CRS cases [72]. In 2008, two studies on men, women of childbearing age, and pregnant women [30,73] from the Banat region (Table 2) reported a prevalence of 94.1%/94.1% between 2008 and 2010 and 91.4%/91.5% between 2015 and 2018, which shows a concerning decrease in the vaccine coverage. A second rubella outbreak was reported in Romania between 2011 and 2012, with a total of 24,627 cases (97.7% were not vaccinated) and 28 cases of CRS [25].

#### 4.2.2. Anti-Rubella IgM

The studies reporting anti-rubella IgM antibodies date as early as 2003, when the National Institute of Public Health published the results of a large rubella outbreak that comprised 115,000 cases [72]. The prevalence reported among the whole country’s population at that time was 0.531%. In the same period, another study [74] from the Moldavia region reported a peak of incidence of 10.525% in urban areas and children under 14. Another rubella outbreak that occurred between 2011 and 2012 had a prevalence of 0.029% in a nationwide study [25]. In the same study, from 6182 anti-rubella-IgM-positive cases analyzed, only 28 cases of congenital rubella syndrome were identified, resulting in 11 neonatal deaths and 1 stillbirth. A study in the Banat region from 2008 to 2010 reported a prevalence of 0.479% of acute infection with rubella vs. 0.273% between 2015 and 2018 in women of childbearing age [30], showing a more than twofold decrease in prevalence. During 2019–2020, the Eurostat website reports a marked decrease in acute rubella, with only four cases in Romania [75], corresponding to a notification rate of 0.2 per 1.000.000 people, and reaching 0 reported cases during 2021–2022 [76].

### 4.3. Cytomegalovirus

#### 4.3.1. Anti-CMV IgG

We found two studies from the Banat region from 1990 [52] and 1993 [51] analyzing pregnant women as well as children, which reported a prevalence of 51%/45% (Table 3). Interestingly, more recent studies, such as that of Gorun et al. from 2020, reported a high prevalence of CMV immunization of 94.6% during 2008–2010 and 91.8% during 2015–2018 [29]; these results are supported by a study on women of childbearing age by Mocanu et al. from 2021 [30], in the absence of immunization by vaccine, as there is no approved vaccine to date. Seroprevalence was found to be higher among women in rural areas compared to those in urban areas [29]. These findings indicate that the western region of Romania has a low risk profile for primary CMV infection during pregnancy, as a significant number of women are seropositive [29]. The development of an anti-CMV vaccine was considered to be a priority by the National Vaccine Program Office of the USA [77].

#### 4.3.2. Anti-CMV IgM

A study from 2006 in women and children from Craiova, Oltenia region showed a prevalence of anti-CMV IgM antibodies of 0.592% [26]. In a study from the Banat area on women of childbearing age, the prevalence was 0.342% in 2008–2010, compared with 0.291% between 2015 and 2018 [30]. We can observe a descending trend of acute infection with CMV in the Banat region.

### 4.4. Herpes Simplex Viruses

#### Anti-HSV IgG

We could only find two studies performed between 2004 and 2005 in the Bucharest area, Muntenia region that assessed the prevalence of IgG antibodies against HSV-1 and HSV-2 in pregnant and non-pregnant women, as well as in men. The prevalence of anti-HSV-1 IgG (87.3%/87.2%) was much higher than that for HSV-2 in both studies (15.1%/15.2%) (Table 4).

The first study was performed on 452 pregnant women aged 15–39 years. The prevalence rates were 87.3% for HSV-1 and 15.1% for HSV-2. The prevalence of HSV-2 seropositivity was influenced by the level of education, being 6.28 times higher in elementary-school graduates and 2.26 times higher in high-school graduates compared with college graduates. Also, the history of sexual partners was directly correlated with the HSV-2 prevalence, which was found to be 2.43 times higher in individuals with two or three partners and 4.26 times higher in those with more than three partners compared to those with one sexual partner [78].

HSV-2 IgG seroprevalence was lower (11.0%) in female teenagers vs. 38.3% in adult women. Also, HSV-2 IgG seroprevalence was increased in women compared with men (17.0% vs. 10.8%). Other risk factors for HSV-2 infection, in addition to female sex and older age, included lower educational status, higher number of sexual partners, and history of genital vesicles [79].

### 4.5. Simultaneous Seroprevalence of IgG Antibodies against TORCH Agents

Simultaneous seroprevalence was observed for IgG antibodies against TORCH agents. A study by Mocanu et al in two separate periods (2008–2010 and 2015–2018) [8] showed that association of IgG anti-*T. gondii* + IgG anti-CMV was detected in 41.4%/36.1% of cases, while that between IgG anti-*T. gondii* + IgG anti-rubella in was detected 41.8%/35.7 of cases. Furthermore, the association of IgG anti-*T. gondii* + IgG anti-CMV + IgG anti-Rubella was 39.6% vs. 33.2%.

### 4.6. Demographic Distribution of Infections with TORCH Agents

The prevalence of antibodies against TORCH agents varied between different regions of Romania. The studies covered various regions of Romania, including the whole country, the Moldavia region, the Oltenia region, and the Banat region. This provides a comprehensive overview of prevalence of TORCH complex infections in different parts of Romania.

#### 4.6.1. Distribution of *T. gondii* Infection in Romania

**Figure 3:**
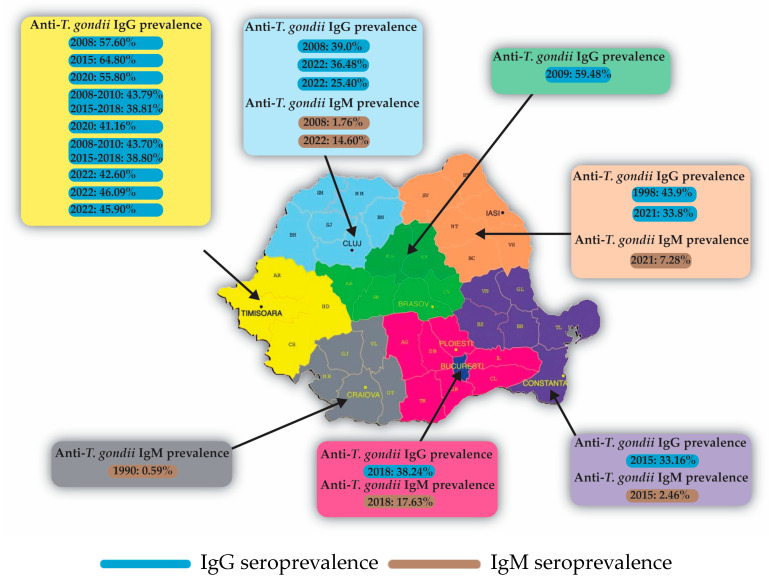
Prevalence of antibodies against *T. gondii* in various regions of Romania: The color of each box corresponds to the color of the geographic region of Romania [14,26,30,50,53,54,55,56,57,58,59,60,61,62,63,64,69].

In 1998, a study performed in the Moldavia region in pregnant women showed a seroprevalence of 43.9% for anti-*T. gondii* IgG [50] (Figure 3). In the same area, another study showed an IgG seroprevalence of 33.84%. The seroprevalence was 33.33% (44/132) in the 25–34 years age group, 40.00% (14/35) in the 36–44 age group, and 29.03% (9/31) in other age groups [61]. The results of a study in the Dobrogea region showed a prevalence of 2.46% for IgM antibodies and 33.16% for IgG antibodies in women aged 15–54 years who had been referred to an infectious diseases hospital with clinical signs of acute toxoplasmosis [14].

In western Romania, we found multiple studies investigating the seroprevalence of anti-*T. gondii* IgG antibodies.

Olariu et al. [62] found a seroprevalence of 57.6% in 2008 in a study performed on 184 consecutive women of childbearing age who presented to a public hospital in Timisoara, Romania. Seroprevalence increased as age increased, from 33.33% in the <20 years age group to 71.4% in the ≥40 years age group. The seroprevalence was higher in rural areas (70%) compared with urban areas (48.1%). The Banat region had different seroprevalence rates, with a study conducted in 2015 showing a rate of 55.55% [54] in women of childbearing age, while other studies conducted in 2020 and later found rates between 38.81% and 55.8% [58,59,63,64].

There were several studies from the Banat region of Romania that compared the seroprevalence of anti-*T. gondii* IgG levels in 2008–2010 with 2015–2018—one performed on pregnant women [59] and another on women of childbearing age [30]. The decrease in prevalence was from 43.79% to 38.81% in the first study [59] and from 43.7% to 38.8% in the second study [30]. The descending trend was maintained for associations between seroprevalence of IgG to multiple TORCH agents.

All of these studies were performed on consecutive patients referred to public hospitals who could be considered to be representative of the population of women of childbearing age. We found another study performed on female blood donors of reproductive age, who are even more suitable as a representative sample of the population, which reported a seroprevalence of 43.2% in female blood donors aged 18–45 years [55].

In the Bihor area, one study reported a low prevalence of 25.4% in pregnant women who were referred to infectious disease clinics [60]. Another study from the same area showed a seroprevalence of 36.48% [64]. These findings suggest that the Bihor area may have a lower prevalence of IgG antibodies compared to the Banat region.

In a broader study performed in northwestern and central Romania, the seroprevalence in the general population was 59.48%, higher in rural areas (63.68%) compared to urban settings (55.12%), with similar rates for both males (60.49%) and females (58.87%) [53].

Also, in the Muntenia region, there was a study on 170 pregnant women that detected anti-*T. gondii* IgG in 38.24% of participants and anti-*T. gondii* IgM in 17.65% of the tested women [57].

In summary, there is considerable variation in the seroprevalence of *T. gondii* in different regions of Romania. The Moldavia region and the Banat region show higher seroprevalence rates compared to the Bihor area. Rural areas generally show higher seroprevalence rates compared to urban areas in the Banat and Bihor areas [30,59,60,64].

We can observe that IgG seroprevalence rates are not very high; conversely, this means that the percentage of non-immunized women is around 60–70%. Therefore, pregnant women can be at risk of acquiring acute toxoplasmosis during gestation, due to the urge of large-scale testing for anti-*T. gondii* antibodies and finding mechanisms to allow for the screening of all pregnant women.

#### 4.6.2. Distribution of Rubella Infection in Romania

**Figure 4:**
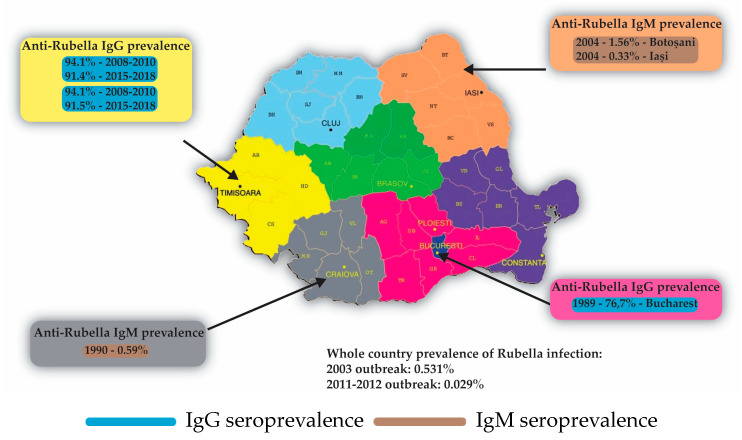
Prevalence of antibodies against rubella in various regions of Romania: The color of each box corresponds to the color of the geographic region of Romania [26,30,49,73,74].

Two studies conducted on men, women of childbearing age, and pregnant women from the Banat region (Table 2, Figure 4) revealed prevalence rates of anti-rubella IgG antibodies of 94.1%/94.1% between 2008 and 2010, and of 91.4%/91.5% between 2015 and 2018 [30,73].

The prevalence of anti-rubella IgM varied significantly between different regions, with 0.531% in the whole country [72] and as high as 1.563% in Botosani County and 0.327% in Iasi County [74] in the Moldavia region.

#### 4.6.3. Distribution of CMV Infection in Romania

**Figure 5:**
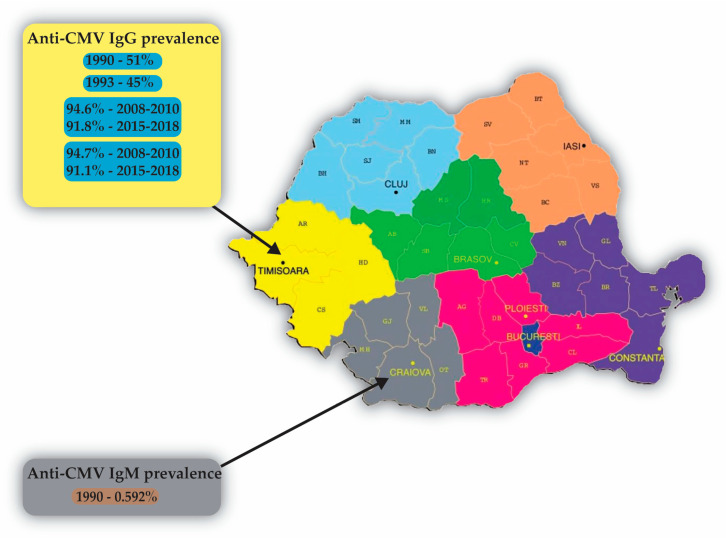
Prevalence of antibodies against CMV in various regions of Romania: The color of each box corresponds to the color of the geographic region of Romania [26,29,30,51,52].

Based on the data collected from the studies (Table 3, Figure 5), it appears that the prevalence of cytomegalovirus (CMV) infection in Romania varies depending on the region and population studied (Figure 5). In the Banat region, studies have found a prevalence of anti-CMV IgG ranging from 45% to 94.7% [29,30]. One study conducted in Craiova, Oltenia region found a prevalence of anti-CMV IgM antibodies of 0.592% [26].

#### 4.6.4. Distribution of HSV Infection in Romania

We could only find two studies from 2010 that investigated the seroprevalence of HSV IgG antibodies among young individuals in Bucharest, Muntenia region. The results revealed higher HSV-2 rates linked to lower educational attainment and increased number of sexual partners. These factors may contribute to a greater risk of HSV-2 transmission within this population.

## 5. Limitations

Our review has some important limitations, in addition to those discussed in the “Quality assessment” section. The studies presented have limited comparability, because of the heterogeneity in the studied groups.

The differences in seroprevalence rates can be explained by confounding due to variables that were unaccounted for, or due to differences between the studied groups. The data were collected from different testing centers and in different time periods. There are significant differences in the populations of individuals who present to public healthcare facilities—where sometimes the tests are conducted for free, based on health insurance—and private testing facilities where people must pay for the tests (selection bias). The differences in population structure on variables like age, socioeconomic status, education level, and other unaccounted variables may confound the studies’ results.

We did not analyze other risk factors linked to toxoplasmosis (such as raw/undercooked meat consumption) that were analyzed in other studies [14]. The seroprevalence of IgM antibodies can be higher in patients who have been referred to the laboratory for confirmation of clinical signs of toxoplasmosis [14]. Also, the studies were performed in different periods, and it is possible that the seroprevalence changes over time.

The laboratory methods used to detect antibodies also differed between the studies, and serological tests may have different sensitivities and specificities. Several studies used latex agglutination to detect the antibodies [55,62], while other studies used immunoenzymatic methods [30,61]. The manual assays have their drawbacks because of timing, contamination risks, and environmental conditions that influence the speed of enzymatic reactions. Modern laboratories use automated analyzers that use thermostats, run internal controls, and adjust the readings to achieve accurate results [80].

## 6. Future Directions

This review brings together prevalence data of infections with agents included in the TORCH complex. These data can offer support for developing national strategies for preventing congenital infections. Inclusion of TORCH antibody tests in the national screening protocols would allow for the early detection of these infections and improve mother–child healthcare, because in Romania there are many mothers who cannot afford proper pregnancy monitoring, which contributes to the high rate of infant mortality [81].

## 7. Conclusions

This study examined the seroprevalence of antibodies against TORCH agents in Romania, uncovering variations by region, age, and demographic factors, with high prevalence of chronic toxoplasmosis (59.48%) in Moldavia and Banat, as well as in the whole of northwestern and central Romania [53], and with anti-*T. gondii* IgG antibodies more prevalent in rural areas and among individuals aged over 40 years. It also highlights the impact of the anti-rubella vaccine on anti-rubella IgG antibodies prevalence, although there has been a recent decrease in vaccination coverage. Instances of simultaneous seroprevalence against multiple TORCH agents were noted, suggesting common risk factors. The findings emphasize the need for ongoing monitoring and targeted interventions, such as vaccination and educational campaigns specifically focusing on pregnant women, to manage the diverse landscape of TORCH infections in Romania.

## Figures and Tables

**Table 1:** Studies included in the review assessing the prevalence of toxoplasmosis in Romania.

Nr	Name	Title	Sample	Prevalence/ Testing Method	Main Findings	Area
1.	Crucerescu E, 1998 [50]	Epidemiological Data On Toxoplasmosis. The Aspects Of Congenital Toxoplasmosis.	224 pregnant women with pathological pregnancies, 347 apparently healthy pregnant women, 1422 newborns, 223 children with mental retardation and visual pathology, 129 apparently healthy children.	43.9% anti-*T. gondii* IgG by indirect immunofluorescence, direct agglutination, and immunosorbent agglutination assay.	The prevalence of congenital toxoplasmosis in the first trimester was 0.6%; 7.1% of the spontaneous abortion cases had anti-*T. gondii* antibodies. The seroprevalence of anti-*T. gondii* IgG in children with intellectual disability was 66.4%, and in those with visual pathology it was 37.4%. In healthy children the seroprevalence was 9.3%.	Moldavia region.
2.	Costache et al., 2008 [56]	Toxoplasmic Infection In Pregnant Women From Cluj County And Neighboring Areas.	68 mothers with clinical and radiological signs of toxoplasmosis.	39.0% anti-*T. gondii* IgG by manual enzyme-linked immunosorbent assay (ELISA).	Toxoplasmic infection was predominantly observed during the first trimester of pregnancy, accounting for 66.66% of cases and representing the primary cause of abortion. The annual infection risk for women aged 20–33 years in our geographic region was determined to be 0.67%. Around 4% of cases displayed IgM persistence for over a year, while an additional 7% exhibited both positive IgM and positive IgG, suggesting a potential persistence in 11% of cases.	Cluj County.
3.	Olariu et al., 2008 [62]	Prevalence Of *Toxoplasma Gondii* Antibodies Among Women Of Childbearing Age In Timis County.	184 consecutive women, aged between 14 and 45 years, who presented to a public hospital in Timisoara, Romania.	57.6% anti-*T. gondii* IgG, tested through latex agglutination.	Seroprevalence increased as age increased from 33.33% in the <20 years age group to 71.4% in the ≥40 years age group. The seroprevalence was higher in rural areas (70%) compared with urban areas (48.1%).	Banat region.
4.	Coroiu et al., 2009 [53]	Seroprevalence Of Anti-*Toxoplasma Gondii* Antibodies In The Healthy Population From Northwestern And Central Romania.	1155 healthy people (males and females) representative of the population in 11 counties from northwestern and central Romania.	59.48% anti-*T. gondii* IgG by manual ELISA.	The seropositivity rate was found to be higher in rural areas (63.68%) compared to urban settings (55.12%), with similar rates for both males (60.49%) and females (58.87%). Seropositivity increased with age. The estimated annual infection risk associated with age showed high susceptibility from childhood to adolescence (3–4.5% annual infection risk), a stable level for adults (1–2%), and then an increase with age (up to 5.5% for individuals 65 years old). In a sample of 105 women aged between 16 and 35 years, the seroprevalence was determined to be 51.43%.	Northwestern and central Romania.
5.	Olariu et al., 2015 [54]	Seroprevalence Of *Toxoplasma Gondii* In Western Romania.	304 individuals.	64.8% anti-*T. gondii* IgG in the general population and 55.55% in women of reproductive age by latex agglutination.	There was no statistically significant disparity in seroprevalence observed between male (60.4%, 84/139) and female (68.5%; *p* < 0.140) participants. A markedly elevated seroprevalence was identified in individuals within the age ranges of 40–49 years (66.7%), 50–59 years (65.5%), 60–69 years (68.6%), and 70–84 years (76.8%) in comparison to those aged 15–19 years (35.0%; *p* = 0.03, *p* < 0.01, *p* < 0.01, and *p* < 0.001, respectively). The overall prevalence of *T. gondii* antibodies among women of reproductive age (16–49 years) was determined to be 55.5%. Furthermore, variations in prevalence were noted in relation to the residential area, affecting both genders.	Banat region.
6.	Rugină et al., 2015 [14]	Toxoplasmosis In Immunocompetent And Immunocompromised Populations Of Constanţa, Romania.	412 adult patients (193 women aged 15–54 years); 15 children aged 0–15 years.	2.46% anti-*T. gondii* IgM and 33.16% IgG in women aged 15–54 years, and 0% IgM and 33.33% IgG in children aged 0–15 years, by enzyme immunoassay sandwich method with a final fluorescence detection (ELFA).	The multivariate analysis conducted on all individuals revealed that several factors were associated with an increased risk of *T. gondii* infection. These factors included consuming undercooked meat (*p* = 0.0001), assessing the taste of meat during cooking (*p* = 0.0002), drinking unpasteurized milk (*p* = 0.0382), and consuming untreated water (*p* = 0.3944).	Dobrogea region.
7.	Neagoe et al., 2018 [57]	Serological And Molecular Diagnosis Of *Toxoplasma Gondii* Infection In Pregnant Women In Romania.	170 pregnant women.	17.65% anti-*T. gondii* IgM by ELISA; 38.24% anti-*T. gondii* IgG.	Out of the 30 women who were positive for both IgM and IgG, only 13.3% presented all indications (positive specific IgM and IgA antibodies, low IgG avidity, and positive PCR) for maternally acquired *T. gondii* infection during pregnancy, posing a risk of congenital transmission. Additionally, a significant association was observed between toxoplasmosis and the consumption of undercooked meat (*p* < 0.05).	Muntenia region.
8.	Căpraru et al., 2019	Toxoplasmosis Seroprevalence In Romanian Children.	441 children aged 1–18 years.	16.6% anti-*T. gondii* IgG.	18.4% rural seroprevalence and 14.7% urban seroprevalence of anti-*T. gondii* IgG antibodies.	
9.	Olariu et al., 2020 [58]	Seroprevalence And Risk Factors Of *Toxoplasma Gondii* Infection In Pregnant Women From Western Romania.	208 pregnant women.	55.8% anti-*T. gondii* IgG by electrochemiluminescence immunoassay (ECLIA).	A diminished educational level and occupational involvement in meat handling were identified as risk factors for *T. gondii* seropositivity. The seroprevalence of *T. gondii* was observed to be higher among pet owners (specifically, cats and/or dogs) compared to individuals without pets (*p* = 0.032). Females with a history of four or more live births exhibited a higher frequency of *T. gondii* seropositivity than those who had no previous births (*p* < 0.002). Additionally, women who experienced spontaneous abortions demonstrated a greater prevalence of *T. gondii* seropositivity than those without such a history (*p* = 0.036).	Banat region.
10.	Motoi et al., 2020 [59]	A Decreasing Trend In Toxoplasma Gondii Seroprevalence Among Pregnant Women In Romania—Results Of A Large-Scale Study.	6889 pregnant women: 1457 between 2008 and 2010, and 5432 between 2015 and 2018.	43.79/38.81% anti-*T. gondii* IgG by chemiluminescent immunoassay (CLIA) and chemiluminescent magnetic microparticle immunoassay (CMIA).	There was a descending trend in *T. gondii* seroprevalence among pregnant women in the western region of Romania, from 43.79% to 38.81% from 2008–2010 to 2015–2018. This trend was consistent in both urban (40.53% vs. 34.85%) and rural areas (52.22% vs. 46.22%), with rural areas having a higher prevalence rate than urban areas. Moreover, there was an ascending trend of seroprevalence with advancing maternal age.	Timisoara County.
11.	Mihu et al., 2020 [63]	Seroprevalence Of *Toxoplasma Gondii* Infection Among Women Of Childbearing Age In An Endemic Region Of Romania, 2016–2018.	2626 childbearing-age women.	41.16% anti-*T. gondii* IgG by CLIA.	The frequency of occurrence of the condition demonstrated an upward trend as a function of advancing age, with rates increasing from 32% in females aged 15–19 years to 62% in females aged 40–45 years. Notably, rural regions exhibited a higher incidence rate, with 46% of the population affected, in contrast to urban areas, where the prevalence was 36%.	Banat region.
12.	Ivănescu et al., 2021 [61]	Toxoplasmosis—A Disease With High Epidemiological Risk In Humans And Animals.	208 women tested for IgM antibodies; 198 women tested for IgG antibodies.	7.21% anti-*T. gondii* IgM And 33.84% anti-*T. gondii* IgG by ELISA.	Of the 15 IgM-positive women, 10 were in the 25–34 years age group, 4 in the 35–44 years age group, and 1 in the 15–19 years age group. The 67 IgG-positive cases were equally scattered across the age groups 0–12 months, 15–19 years, 20–24 years, 25–34 years, 35–44 years, 45–54 years, and 55–64 years.	Moldavia region.
13.	Mocanu et al., 2021 [30]	Simultaneous Seroprevalence Of *Toxoplasma Gondii*, Cytomegalovirus, And Rubella Virus In Childbearing Women From Western Romania.	6961 women of childbearing age during two successive intervals: 2008–2010 (n = 1461) and 2015–2018 (n = 5500).	43.7%/38.8% anti-*T. gondii* IgG and 0.821%/1.054% anti-*T. gondii* IgM by CLIA.	Association of IgG anti-*T. gondii* + IgG anti-CMV—41.4% vs. 36.1%, OR = 0.79, *p* = 0.0002. Association of IgG anti-*T. gondii* + IgG anti-rubella—41.8% vs. 35.7%, OR = 0.77, *p* < 0.0001. Association of IgG anti-*Toxoplasma* + IgG anti-CMV + IgG anti-rubella—39.6% vs. 33.2%, OR = 0.75, *p* < 0.0001.	Banat region.
14.	Mocanu et al., 2022 [69]	The Impact Of Latent *Toxoplasma Gondii* Infection On Spontaneous Abortion History And Pregnancy Outcomes: A Large-Scale Study.	806 pregnant women.	42.6% anti-*T. gondii* IgG by CLIA.	There was no observed correlation between a history of latent *T. gondii* infection and spontaneous abortion. This finding held true regardless of whether the analysis compared the number of women who experienced spontaneous abortion to those with at least one prior pregnancy, or if it considered the total count of spontaneous abortions in comparison to the total number of pregnancies in both groups (OR = 1.288/1.156, *p* = 0.333/0.536).	Banat region.
15.	Csep et al., 2022 [60]	Research On Demographic, Clinical, And Paraclinical Aspects In Pregnant Women Infected With *Toxoplasma Gondii*.	240 pregnant women with clinical signs of toxoplasmosis.	25.4% anti-*T. gondii* IgG and 14.6% anti-*T. gondii* IgM by CLIA.	No statistically significant disparities were observed in the distribution of pregnant women across age groups (*p* = 0.0997), by years and age groups (*p* = 0.4410), or by region of origin (*p* = 0.3105). Additionally, there was no significant difference in distribution by region of origin and year of study (*p* = 0.4804).	Bihor County.
16.	Mihu et al., 2022 [64]	Seroprevalence Of *Toxoplasma Gondii* In Females Aged 15–45 Years From Bihor County, Western Romania.	1935 females aged 15–45 years.	36.48% anti-*T. gondii* IgG by CLIA.	The prevalence of *T. gondii* IgG-positive females demonstrated an inclination to escalate with advancing age. A higher seroprevalence was observed among females residing in rural regions (47.79%) in comparison to those inhabiting urban areas (30.95%).	Bihor County.
17.	Mihu et al., 2022 [66]	Screening For The Detection Of *Toxoplasma Gondii* Igg, Igm, And Iga In Females Of Reproductive Age From Western Romania.	1317 women aged 15–45 years.	46.90% anti-*T. gondii* IgG and 8.90% anti-*T. gondii* IgM by CLIA.	The prevalence of IgG tended to increase with age. The prevalence of IgA was 1.65%. The study concluded that IgA antibodies are rarely detected in serological screening, but in subjects positive for both IgG and IgM antibodies the IgA test can raise the rate of detection of recent *T. gondii* infection.	Bihor County.
18.	Lupu et al., 2022 [55]	Seroepidemiology Of *Toxoplasma Gondii* Infection In Blood Donors From Western Romania.	1347 healthy blood donors.	45.9% anti-*T. gondii* IgG detected by latex agglutination; 43.2% anti-*T. gondii* IgG in female blood donors aged 18–45 years.	The prevalence of anti-*T. gondii* IgG antibodies increased with age from 32.6% in the 18–25 years age group to 67.6% in the 56–63 years age group. The seroprevalence increased as the education level decreased. The multivariate analysis detected significant associations between seroprevalence and owning pets, age, and education level	Banat region.

**Table 2:** Studies included in the review assessing the prevalence of rubella in Romania.

Nr	Name	Title	Sample	Prevalence/Testing Method	Main Findings	Area
1.	Dumitrescu et al., 1989 [49]	Evaluation Of The Anti-Rubella Immunity Levels In A Lot Of 5000 Sera From Women At Procreative Age, Tested By HAI, In Romania.	5030 women aged 15–40 years.	Anti-*T. gondii* IgG seroprevalence was 76.70% according to hemagglutination inhibition (HAI).	The seroprevalence was 79.5% in women aged 15–20 years and 75.3% in the 31–35 years age group.	Whole country.
2.	Rafila et al., 2003 [72]	A Large Rubella Outbreak, Romania, 2003.	115,000 cases.	0.531% anti-Rubella IgM.	The vaccinated adolescents had significantly lower incidence rates of acute rubella infection (*p* < 0.001).	Whole country.
3.	Costan et al., 2004 [74]	Epidemiological Research On Rubella Incidence In Various Demographic Structures, In A Northeastern Area Of Romania.	4895 cases.	1.563% (Botosani County)/0.327% (Iasi County) anti-rubella IgM by ELISA.	The peak incidence was 10.525% in 2003, predominantly in urban areas; also, the incidence was higher in children under 14. The incidence in males was 0.536%.	Moldavia region.
4.	Neamtu et al., 2006 [26]	Clinical–Statistical Study Of Patients Who Suffer From Infections That Are Brought About Factors Of The TORCH Complex.	169 women and children.	0.592% anti-rubella IgM by ELISA.	The prevalence was higher in urban areas.	Craiova.
5.	Lazar et al., 2016 [25]	Epidemiological And Molecular Investigation Of A Rubella Outbreak, Romania, 2011 To 2012.	24,627 cases (clinical criteria), out of which 6182 were anti-rubella-IgM-positive.	0.029% anti-rubella IgM by ELISA.	Rubella cases predominantly affected individuals between the ages of 1 and 65, with a median age of 18 years. The age group most affected was 15 to 19 years, with a total of 16,245 reported cases. Almost all of the cases (97.7%) reported no previous history of vaccination, amounting to 24,067 cases. Out of the total number of cases reported, 28 cases of congenital rubella syndrome (CRS) were identified, resulting in 11 neonatal deaths and one stillbirth.	Whole country.
6.	Gorun et al., 2021 [73]	Prevalence Of Rubella Antibodies Among Fertile Women In The West Of Romania, 18 Years After The Implementation Of Immunization.	1452 individuals between 2008 and 2010, and 5462 between 2015 and 2018.	94.1% (92.7–95.2) versus 91.4% (90.6–92.1; OR = 0.76; *p* = 0.0007) anti-rubella IgG by CLIA.	Based on the birth year and eligibility for the vaccination program, RV seroprevalence rates were 82.4% (76.8–86.8) for 1997–2004, 85.4% (80.5–89.3) for 1995–1996, 90.1% (89.0–91.1) for those born before 1989, and 95.8% (94.7–96.6) for 1989–1994. No significant differences in RV seropositivity were observed based on the place of residence. Overall RV susceptibility increased from 2008–2010 to 2015–2018. The highest susceptibility was found among women born between 1997 and 2004 who were eligible for the measles–mumps–rubella (MMR) vaccine through the family practice system, while the lowest susceptibility was found among women born between 1989 and 1994 who were eligible for the monovalent rubella vaccine administered in schools.	Banat region.
7.	Mocanu et al., 2021 [30]	Simultaneous Seroprevalence Of *Toxoplasma Gondii*, Cytomegalovirus, And Rubella Virus In Childbearing Women From Western Romania.	6961 women of childbearing age during two successive intervals: 2008–2010 (n = 1461) and 2015–2018 (n = 5500).	94.1%/91.5% anti-rubella IgG by CLIA; 0.479%/0.273% anti-rubella IgM.	Association of IgG anti-*T. gondii*/IgG anti-rubella—41.8% vs. 35.7%, OR = 0.77, *p* < 0.0001. Association of IgG anti-CMV/IgG anti-rubella—88.9% vs. 83.6%, OR = 0.63, *p* < 0.0001. Association of IgG anti-*T. gondii* and IgG anti-CMV/IgG anti-rubella—39.6% vs. 33.2%, OR = 0.75, *p* < 0.0001.	Banat region.

**Table 3:** Studies included in the review assessing the prevalence of Cytomegalovirus infection in Romania.

Nr	Name	Title	Sample	Prevalence/ Testing Method	Main Findings	Area
1.	Rosiu et al., 1990 [52]	A Serological Probe Of Cytomegalic Infection In Southwestern Romania. I. The Presence Of Igg-CMV In A Sample Healthy Population.	374 pregnant women.	51% anti-CMV IgG by hemagglutination.		Banat region.
2.	Moldovan et al., 1993 [51]	The Epidemiological Aspects Of Cytomegalic Infection In The Banat Region, Romania.	711 adults and children.	45% anti-CMV IgG by hemagglutination.		Banat region.
3.	Neamtu et al., 2006 [26]	Clinical–Statistical Study Of Patients Who Suffer From Infections That Are Brought About Factors Of The TORCH Complex.	169 women and children.	0.592% anti-CMV IgM by ELISA.	The prevalence was higher in urban areas, due to easier access to medical services.	Craiova city.
4.	Gorun et al., 2020 [29]	Cytomegalovirus Seroprevalence In Pregnant Women In The Western Region Of Romania: A Large-Scale Study.	8951 pregnant women during two successive intervals: 2008–2010 (n = 1466) and 2015–2018 (n = 7485).	94.6%/91.8% anti-CMV IgG by CLIA.	Seroprevalence was found to be higher among women in rural areas compared to those in urban areas. The findings indicate that the western region of Romania has a low risk profile for primary CMV infection during pregnancy, as a significant number of women are seropositive.	Banat region.
5.	Mocanu et al., 2021 [30]	Simultaneous Seroprevalence Of *Toxoplasma Gondii*, Cytomegalovirus, And Rubella Virus In Childbearing Women From Western Romania.	6961 women of childbearing age during two successive intervals: 2008–2010 (n = 1461) and 2015–2018 (n = 5500).	94.7%/91.1% anti-CMV IgG 0.342%/0.291% anti-CMV IgM by CLIA.	Simultaneous seropositivity for *Toxoplasma gondii* and rubella virus decreased from 2008–2010 to 2015–2018, but the susceptibility to these infections increased.	Banat region.

**Table 4:** Studies included in the review assessing the prevalence of HSV infection in Romania.

Nr	Name	Title	Sample	Prevalence/ Testing Method	Main Findings	Area
1.	Arama et al., 2008 [78]	Seroprevalence And Risk Factors Associated With Herpes Simplex Virus Infection Among Pregnant Women.	452 pregnant women, aged 15–39.	87.3% anti-HSV-1 and 15.1% anti-HSV-2 IgG by ELISA.	Elementary school: RR = 6.28, high school: RR = 2.26 compared with college graduates. Presence of 2–3 sexual partners: RR = 2.43 or >3 partners: RR = 4.26 compared with only 1 sexual partner.	Bucharest area.
2.	Arama et al., 2010 [79]	Type-Specific Herpes Simplex Virus-1 And Herpes Simplex Virus-2 Seroprevalence In Romania: Comparison Of Prevalence And Risk Factors In Women And Men.	1058 women and men.	87.2% anti-HSV-1 IgG and 15.2% anti-HSV-2 IgG by ELISA.	HSV-1 IgG seroprevalence had no clear trend by age or by sex, but it was increased in people with low educational status and residence in rural areas. HSV-2 IgG seroprevalence was higher (11.0%) in teenagers vs. 38.3% in adults. Also, it was increased in women compared with men (17.0% vs. 10.8%). The risk factors for HSV-2 infection were lower educational status, higher number of sexual partners, and history of genital vesicles.	Bucharest area.

## Data Availability

The data presented in this study are available upon request from the corresponding author. The data are not publicly available due to the patient personal data protection policy of the university and hospital.
